# Targeting Programmed Cell Death to Improve Stem Cell Therapy: Implications for Treating Diabetes and Diabetes-Related Diseases

**DOI:** 10.3389/fcell.2021.809656

**Published:** 2021-12-16

**Authors:** Qi Zhang, Xin-xing Wan, Xi-min Hu, Wen-juan Zhao, Xiao-xia Ban, Yan-xia Huang, Wei-tao Yan, Kun Xiong

**Affiliations:** ^1^ Department of Anatomy and Neurobiology, School of Basic Medical Sciences, Central South University, Changsha, China; ^2^ Department of Endocrinology, Third Xiangya Hospital, Central South University, Changsha, China

**Keywords:** programmed cell death, stem cell, apoptosis, pyroptosis, necroptosis, diabetes

## Abstract

Stem cell therapies have shown promising therapeutic effects in restoring damaged tissue and promoting functional repair in a wide range of human diseases. Generations of insulin-producing cells and pancreatic progenitors from stem cells are potential therapeutic methods for treating diabetes and diabetes-related diseases. However, accumulated evidence has demonstrated that multiple types of programmed cell death (PCD) existed in stem cells post-transplantation and compromise their therapeutic efficiency, including apoptosis, autophagy, necroptosis, pyroptosis, and ferroptosis. Understanding the molecular mechanisms in PCD during stem cell transplantation and targeting cell death signaling pathways are vital to successful stem cell therapies. In this review, we highlight the research advances in PCD mechanisms that guide the development of multiple strategies to prevent the loss of stem cells and discuss promising implications for improving stem cell therapy in diabetes and diabetes-related diseases.

## Introduction

Stem cells (SCs) are unique cell populations distinguished by the capacity of self-renewal and differentiation (Biswas and Hutchins, 2007; McElhinney et al., 2020). These unique features of SCs make them the preferred candidate for tissue repairing (Yang et al., 2019; Yang et al., 2020; Qin et al., 2021). According to different developmental stages, SCs can be categorized into distinct types, such as embryonic SCs (ESCs), induced pluripotent SCs (IPSCs), and adult SCs (ASCs) (Bogliotti et al., 2018; Hu et al., 2021). These SCs are widely utilized for regenerative medicine therapies (Gurusamy et al., 2018; Fatima et al., 2019).

The worldwide shortage of pancreas donors remains a major hurdle to islet transplantation, and SC therapy represents a highly promising alternative approach for treatments of advanced diabetes (Saleem et al., 2019; Chen et al., 2020). In SC therapy for type 1 diabetes mellitus (T1DM), insulin-producing cells can be generated from SCs (Manzar et al., 2017; Chen et al., 2020). Neural SCs (NSCs), bone marrow-derived mesenchymal SCs (BM-MSCs), and umbilical cord MSCs (UC-MSCs) are a promising treatment for diabetic retinopathy and foot ulcers (Zhang et al., 2017; Zhao et al., 2020a; Huang Q. et al., 2021).

However, the cell death of SC post-transplantation creates significant challenges to transplantation therapy (Mastri et al., 2014). According to different death processes, cell death are categorized as: programmed cell death (PCD), a precise and genetically controlled cellular death, and non-PCD, also called necrosis (Cheng et al., 2018; Guo LM. et al., 2020; Bedoui et al., 2020). Extensive pharmacological and genetic strategies have been developed to inhibit PCD to prevent cell loss and thus improve physiological function of organs (Wang Z. et al., 2018; Yuan et al., 2019; Wu X. et al., 2020; Yan W.-T. et al., 2021). Increasing evidence indicates a close link between PCDs and cell death of transplanted SCs (Ho et al., 2017; Wang R. et al., 2020; Pierozan et al., 2020). More importantly, targeting these PCDs shows promising therapeutic effects for diabetes and diabetes-related diseases (Zhang K. et al., 2019; Hu et al., 2019).

### Distinct Forms of PCD IN SC for Transplantation

#### Apoptosis

**FIGURE 1:**
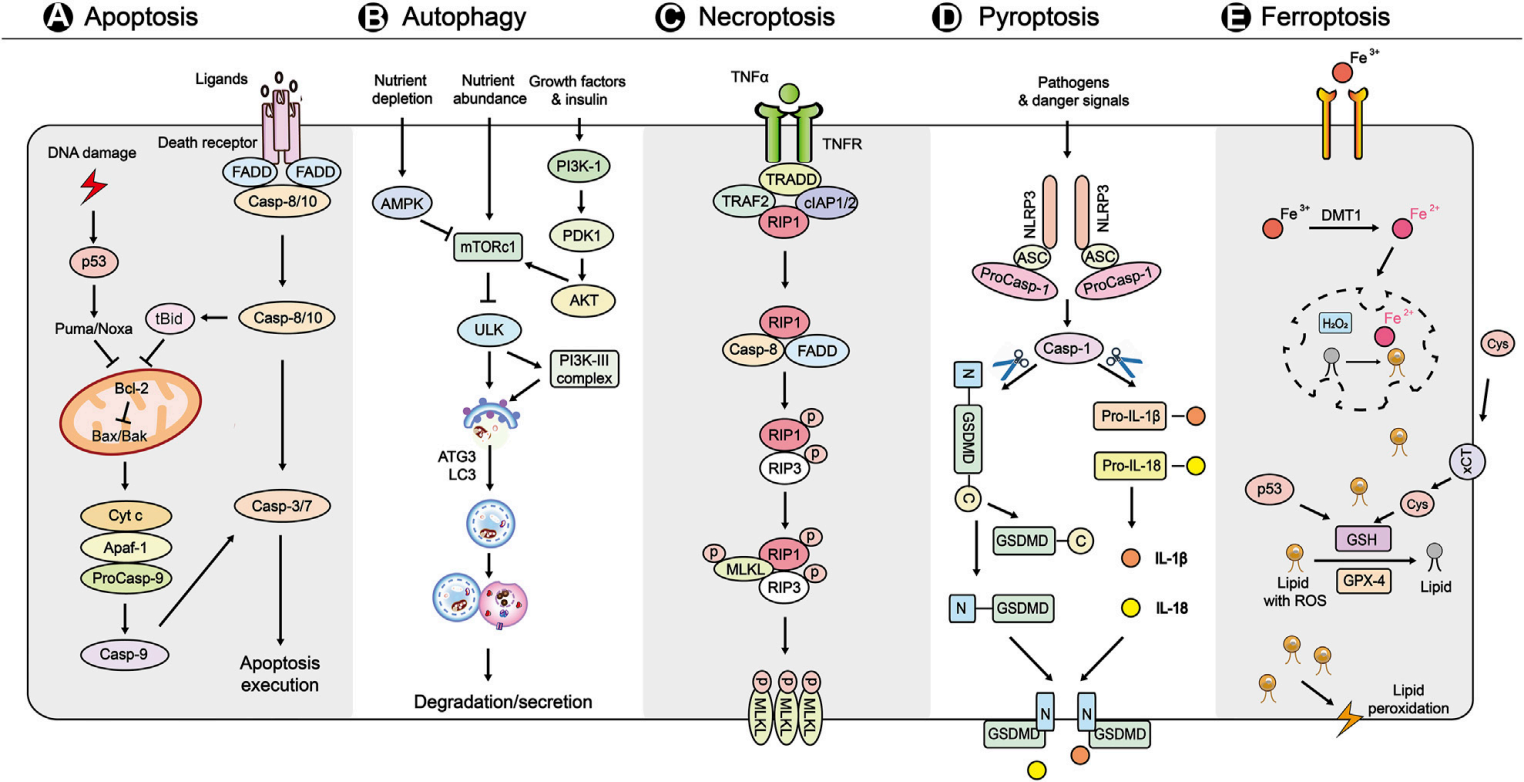
A brief overview of the main molecular mechanisms in PCDs **(A)** Apoptosis is initiated by the intrinsic and extrinsic pathways. In the intrinsic pathway, DNA damage activates p53, and subsequently activates Puma/Noxa to induces signaling genes including proapoptotic proteins (Bax, Bak, tBid), apoptosome (Cyt c, Apaf-1, pro-Caspase-9), antiapoptotic protein (Bcl-2), and apoptosis execution factors (caspase-3/7). The extrinsic pathway of apoptosis is initiated by the binding of TNF to its receptors, leading to the recruitment of FADD and caspase-8. In addition, caspase-8 can cleave Bid to t-Bid, which participates in the intrinsic pathway of apoptosis **(B)** Autophagy is initiated by nutrient sensoring, including AMPK, PI3K-1, mTORC1. Autophagosome formation and maturation are mediated PI3K-III complex, ATG3, and LC3. Finally, the autophagosome contents undergo is degraded by lysosomes **(C)** Necroptosis is initiated by the binding of TNF-α and the receptor TNFR. Under conditions of caspase-8 (initiator caspase of extrinsic apoptosis) is not active, the formation of necroptosome (integration of phosphorylated RIPK1 and RIPK3) induces the MLKL phosphorylation and oligomerization. Finally, the MLKL oligomers translocation to membranes and disrupt it to kill cells **(D)** Pyroptosis is triggered by various pathogens and danger signals. These signals activate NLRP3 inflammasome, which consists of NLRP3, ASC and procaspase-1, and subsequently leads to cleavage of GSDMD and pro-IL-1β and pro-IL-18. Finally, the N-terminal fragment of GSDMD targets to membrane to form membrane pores and induces inflammatory cell death **(E)** Ferroptosis is triggered by severe lipid peroxidation with ROS and iron overload, leading to membrane damage. The uptake of Fe^2+^ is regulated by DMT1. The lipid peroxidation is mainly caused by loss of activity of GSH and GPX4. The xCT also functions in regulating ferroptosis *via* Cys. In addition, p53, an initiator of intrinsic apoptosis, controls ferroptosis by regulation of the production of GSH. Abbreviations: Cyt c, cytochrome c; Apaf-1, apoptotic peptidase activating factor 1; Bcl-2, B cell chronic lymphocytic leukaemia/lymphoma-2; FADD, Fas-associated death domain; AMPK, AMP activated protein kinase; mTORC1, mammalian target of rapamycin complex 1; PI3K-1, phosphatidylinositol 3 kinase-1; ATG3, autophagy-related gene 3; LC3, light chain 3; TNF, tumor necrosis factor; TNFR, TNF receptor; TRAF2, TNF receptor associated factor 2; TRADD, TNFR1-associated death domain protein; cIAP1/2, cell inhibitor of apoptosis protein-1/2; RIPK1/3, receptor interacting protein kinase 1/3; MLKL, mixed lineage kinase domain-like protein; NLRP3, nod-like receptor protein-3; ASC, apoptosis-associated speck-like protein; GSDMD, gasdermin D; IL, interleukin; ROS, reactive oxygen species; DMT1, divalent metal ion transporter 1; xCT, cystine/glutamate antiporter SLC7A11; Cys, cystine; GSH, glutathione; GPX4, glutathione peroxidase 4.

Apoptosis is characterized by the breaking up of cell in apoptotic bodies (Nikoletopoulou et al., 2013). In intrinsic pathway of apoptosis, DNA damage can activate p53, and subsequently induce genes involved in apoptosis signaling and execution (Figure 1A) (Hafner et al., 2019). In human ESCs, the stabilization of p53 can suppress the pluripotency of SCs after DNA damage responses (Zhang et al., 2014). In addition, silencing of the proapoptotic gene *Puma*, which is responsible for p53-dependent apoptosis, can increase pluripotency of iPSCs (Lake et al., 2012; Fu et al., 2020). Moreover, proapoptotic BCL-2 signals and ASPP1, an apoptosis-stimulating protein of p53, contributed to the induction of apoptosis in HSCs (Yamashita et al., 2015; 2016).

The extrinsic pathway of apoptosis is initiated by docking of death ligands of tumor necrosis factor (TNF) to TNF receptors 1 (TNFR1) (Carneiro and El-Deiry, 2020). TNF-α can induce apoptosis in NSCs by upregulating the phosphatidylinositol p38 mitogen-activated protein kinase (p38 MAPK) pathway (Chen et al., 2016). In HSC transplantation for treating malignancies, activation of TNF-α-TNFR1 signaling pathway caused accumulation of reactive oxygen species (ROS) in HSCs and subsequent cell damage (Ishida et al., 2017). In contrast, TNF-α-TNFR2 signaling is important for survival and function of MSCs and endothelial stem/progenitor cells (EPCs), and its deficiency resulted in reduced proliferation rate and diminished immunomodulatory effect of these cells (Beldi et al., 2020a; Beldi et al., 2020b; Naserian et al., 2020; Nouri Barkestani et al., 2021; Razazian et al., 2021).

### Autophagy

Autophagy is a self-degradative process that contributes to removing excessive or misfolded proteins and clearing damaged organelles at critical times (Figure 1B) (Glick et al., 2010; Andrade-Tomaz et al., 2020). The autophagy is triggered by upregulation of AMP activated protein kinase (AMPK) and downregulation of mammalian target of rapamycin complex 1 (mTORC1) (Kim et al., 2011). In ESCs and HSCs, the regulation of AMPK and mTOR kinase is essential to their homeostasis, self-renewal and pluripotency (Huang et al., 2009; Gong et al., 2018; Suvorova et al., 2019). Additionally, the precise regulation of mTOR by Sox2 is vital to reprogramming of somatic cells to form iPSCs (Wang S. et al., 2013). The viability and stemness of NSCs and ESCs were also associated with LC3 lipidation, autophagic flux, and formation of autophagosomes (Bialik and Kimchi, 2010; Vázquez et al., 2012; Gu et al., 2019; Wang et al., 2019). Additionally, the autophagy-related gene *ATG3* was shown to be a pivotal regulator of mitochondrial homeostasis regulation in ESCs (Liu et al., 2016).

Notably, Dou *et al.* demonstrated that an amyloid binding peptide with three chaperone-mediated autophagy motifs significantly reduced Aβ oligomers in iPSC cortical neurons (Dou et al., 2020). Autophagy driven by FOXO3A and FOXO1 also protected HSCs from metabolic stress and guarded ESC identity (Warr et al., 2013; Liu et al., 2017). More importantly, the coordination of autophagy and apoptosis is vital to maintaining homeostasis in BM-MSCs (Zhang et al., 2016).

### Necroptosis

Necroptosis is a programmed form of necrosis mediated by receptor interacting protein kinase 1/3 (RIPK1/3) and mixed lineage kinase domain-like (MLKL) proteins (Figure 1C) (Wang M. et al., 2020; Font-Belmonte, 2020; Yan WT. et al., 2021; Liao et al., 2021). Necroptosis of intestinal SCs triggered bowel inflammation in the pathogenesis of inflammatory bowel disease (Wang R. et al., 2020). In addition, compression triggered necroptosis of nucleus pulposus-derived SCs and inhibiting necroptosis rescued regeneration of degenerated intervertebral discs (Hu B. et al., 2020). Furthermore, inhibition of necroptosis is a novel strategy for allogeneic HSCs transplantation and spermatogonial SC-based therapy for male fertility preservation (Matsuzawa-Ishimoto et al., 2017; Xie et al., 2020). Moreover, cellular transplant therapy based on human olfactory SCs ameliorated motor function in Huntington’s disease by preventing necroptosis (Bayat et al., 2021). Intriguingly, TNF-α also could function as a pro-regeneration factor in HSCs that primarily prevented necroptosis rather than apoptosis by activating a p65-nuclear factor κB-dependent gene program (Yamashita and Passegué, 2019).

### Pyroptosis

Pyroptosis is specific PCD mediated by cleavage of gasdermin D (GSDMD) to form membrane pores and activation of cytokines (Figure 1D) (Chu et al., 2020; Chen Y. et al., 2021; Huang Y. et al., 2021). Pyroptosis contributed to the cell death of human cardiac SCs (hCSCs) in an acute hyperglycemic microenvironment, which impaired cardiac regeneration in diabetic hearts (Yadav et al., 2020). The culture media collected from pyroptotic bone marrow-derived macrophages also induced pyroptosis in MSCs (Zhang C. et al., 2020). Additionally, the pyroptosis of neural progenitor cells represented a therapeutic target in Zika virus-induced brain atrophy (He Z. et al., 2020). Moreover, chitosan thermosensitive hydrogel enhanced the therapeutic efficacy of BM-MSCs for myocardial infarction by alleviating pyroptosis of vascular endothelial cells (Liu Y. et al., 2020). Finally, emerging evidence showed that SCs and SC-derived exosomes inhibited pyroptosis and could be used to treat different diseases (Zhang J. et al., 2020; Yan et al., 2020; Chen M.-T. et al., 2021).

### Ferroptosis

Ferroptosis is an iron-dependent form of cell death (Figure 1E) (Stockwell et al., 2017). The iPSC-derived cell modeling of neuroferritinopathy revealed that iron-dependent ferroptosis has a primary role in neuronal aging and degeneration (Cozzi et al., 2019). Also, iron overload (IOL) may induce cellular toxicity in hematopoietic SCs therapy for hematologic malignancies, and IOL reduction may improve outcomes (Leitch et al., 2017). In addition, catecholic flavonol quercetin inhibited erastin-induced ferroptosis in BM-MSCs (Li et al., 2020). Moreover, in Pelizaeus-Merzbacher disease, the mutant oligodendrocytes of patients exhibited the hallmarks of ferroptosis, and gene correction in patient-derived iPSCs rescued the iron-induced cell death (Nobuta et al., 2019).

The characteristics of distinct kinds of PCD in transplanted SCs provide an in-depth understanding of cell death in SC therapy. Based on the key mediators and crosstalk identified in PCD, the development of highly precise strategies to improve SC survival is possible.

### Which Types of PCD Reported IN SC can be Used to Treat Diabetes and Diabetes-Related Diseases?

Whereas SC therapy represents a highly promising therapeutic strategy for treating diabetes, PCD existed in SCs hinders the therapeutic effects (Saleem et al., 2019). For example, hyperglycemia increased apoptosis of Adipose-derived SCs (ADSCs) and decreased their paracrine function in diabetic retinopathy (Hajmousa et al., 2016; Xu et al., 2020). Furthermore, BM-MSCs from streptozotocin-induced diabetic rats showed impaired antiapoptosis, proliferation and paracrine abilities (Jin et al., 2010).

More importantly, overexpression of hypoxia-inducible factor 1α (HIF1α), a regulator of oxygen homeostasis, significantly alleviated the ADSC apoptosis rate and enhanced diabetic wound closure (Xu et al., 2020). Norepinephrine can also reverse high glucose-induced apoptosis in MSCs through the AKT/BCL-2 pathway (Kong et al., 2019). The peroxisome proliferator-activated receptor- γ agonist pioglitazone (PGZ) is used for management of diabetes (Cho et al., 2019). It was reported that PGZ had a protective effect on compression-mediated apoptosis in MSCs by suppressing mitochondrial apoptosis pathway (Hu et al., 2019).

In addition, autophagy played a protective role in ADSC under high glucose stress (Li et al., 2018). More importantly, the overexpression of Aurora kinase A (AURKA), a cell cycle-regulated kinase, enhanced autophagy of ADSCs, decreased apoptosis, and promoted wound healing in diabetic mice (Yin et al., 2020). Also, the inhibition of autophagy significantly promoted high glucose/ROS-mediated apoptosis in ADSCs (Li et al., 2018). Furthermore, upregulating autophagy in periodontal ligament SCs partially recovered periodontium tissues in a diabetic rat periodontal trauma model, suggesting the protective role of autophagy for SC transplantation (Zhang K. et al., 2019). Additionally, exosomes derived from MSCs ameliorated type 2 diabetes by activating autophagy *via* AMPK pathway (He Q. et al., 2020). Moreover, pyroptosis contributed to the cell death of hCSCs in an acute hyperglycemic microenvironment, which impaired cardiac regeneration in diabetic hearts (Yadav et al., 2020).

Although emerging evidence indicates that many forms of PCD play vital roles in the cell death of SCs for treating diabetes and diabetes-related diseases, the identification of necroptosis and ferroptosis remains to be explored further.

### Current Strategies to Prevent PCD OF SC for Transplantation

#### Preconditioning

The benefit of preconditioning of SC was first described in ischemic myocardium, and to date, a variety of preconditioning strategies have been shown to improve SC survival (Table 1) (Sart et al., 2014; Hu and Li, 2018). Hypoxic preconditioning could decrease apoptosis and increased autophagy in MSCs and BM-MSCs (Liu et al., 2013; Liu et al., 2015a; Lan et al., 2015). Exposure to oxidative stress decreased apoptosis of BM-MSCs upon serum withdrawal and oxidative stress (Pendergrass et al., 2013; Guo L. et al., 2020). Furthermore, heat shock pretreatment enhanced repair effects of MSCs for acute lung injury and premature ovarian failure *via* reducing apoptosis and macrophages (Chen et al., 2018; Lv et al., 2021).

**TABLE 1:** Current strategies to prevent PCD of SC for transplantation.

Strategy	Method	Targeting PCD	SC	Application	References
Preconditioning					
Hypoxia	1% O_2_ for 48 h	Apoptosis	AD-MSCs	Tissue regeneration	Liu et al. (2013)
	1.5% O_2_ for 24 h	Apoptosis	MSCs	Idiopathic pulmonary fibrosis	Lan et al. (2015)
	5% O_2_ for 6 h	Apoptosis; autophagy	BM-MSCs	Diabetic lower-limb ischemia	Liu et al. (2015a)
	5% O_2_ for 48 h	Apoptosis	BM-MSCs	Diabetic lower-limb ischemia	Liu et al. (2015b)
Oxidative stress	100 mM H_2_O_2_ for 2 days	Apoptosis	Cardiac progenitor cells	Heart failure	Pendergrass et al. (2013)
	50 μM H_2_O_2_ for 12 h	Apoptosis	BM-MSCs	Wound healing	Guo et al. (2020b)
Heat shock	42 C for 1 h	Apoptosis	UC-MSCs	Acute lung injury	Lv et al. (2021)
	42 C for 1 h	Apoptosis	BM-MSCs	Premature ovarian failure	Chen et al. (2018)
Lipopolysaccharide	1.0 l g/mL for 24 h	Apoptosis	BM-MSCs	Hypoxia and serum deprivation	Wang et al. (2013a)
Melatonin	5 μM for 24 h	Apoptosis	BM-MSCs	Ischemic kidney	Mias et al. (2008)
Oxytocin	10 nM for 24 h	Apoptosis	BM-MSCs	Hypoxia and serum deprivation	Noiseux et al. (2012)
Sevoflurane	2 vol% for 2 h	Apoptosis	BM-MSCs	Hypoxia and serum deprivation	Sun et al. (2014)
Resveratrol	10 µM for 10 h	Autophagy	ESCs	Enhancing pluripotency of SC	Suvorova et al. (2019)
	10 µM for 2 h	Apoptosis	ADSC	Type 1 diabetes	Chen et al. (2019b)
**Genetic modification**					
AURKA	Lentivirus vectors transfection	Apoptosis; autophagy	ADSC	Diabetic wound healing	Yin et al. (2020)
VEGF_165_	Bi-Tet transfection	Apoptosis	ESCs	Cardiac function	Xie et al. (2007)
HGF	Adenoviral vector transfection	Apoptosis	UC-MSCs	Acute liver failure	Tang et al. (2016)
	Adenoviral vector transfection	Apoptosis	BM-MSCs	Hepatocirrhosis	Zhang et al. (2018)
ERBB4	Lentivirus vectors transfection	Apoptosis	MSCs	Myocardial infarction	Liang et al. (2015)
HIF1α	Lentivirus vectors transfection	Apoptosis	ADSC	Diabetic wound healing	Xu et al. (2020)
	Adenoviral vector transfection	Apoptosis	MSCs	Myocardial infarction	Huang et al. (2014)
**3D cell culturing**					
3D-dynamic system	Culturing for 48 h	Apoptosis	BM-MSCs	Myocardial infarction	Wang et al. (2018a)
3D floating culture	Culturing for 3 days	Apoptosis	MSCs	Enhancing survival of SC	Komatsu et al. (2020)
3D organ culture	Culture with d-serine and RA for 3 weeks	Apoptosis	Spermatogonial SCs	Spermatogenesis	Modirshanechi et al. (2020)
**Co-transplantation**					
NSCs and OECs	NSCs: OECs = 1:1	Apoptosis	NSCs	Traumatic brain injury	Liu et al. (2014)
EPI-NCSCs and OECs	EPI-NCSCs: OECs = 1:1	Apoptosis	EPI-NCSCs	Peripheral nerve injury	Zhang et al. (2019b)
BM-MSCs and monocytes	BM-MSCs: monocytes = 1:30	Apoptosis	BM-MSCs	Facial nerve axotomy	Wu et al. (2020a)

Abbreviations: SCs, stem cells; MSCs, mesenchymal stem cells; ESCs, embryonic stem cells; AD-MSCs, adipose-derived mesenchymal stem cells; BM-MSCs, bone marrow-derived mesenchymal stem cells; UC-MSCs, umbilical cord-derived mesenchymal stem cells; ADSC, Adipose-derived stem cells; NSCs, neural stem cells; EPI-NCSCs, epidermal neural crest stem cells; OECs, olfactory ensheathing cells; RA, retinoic acid; AURKA, Aurora kinase A; VEGF_165_, vascular endothelial growth factor 165; HGF, hepatocyte growth factor; ERBB4, v-erb-b2, avian erythroblastic leukemia viral oncogene homolog 4; HIF1α, hypoxia-inducible factor 1α.

Preconditioning of SCs with pharmacological or chemical agents also improved SCs survival *via* preventing PCD. For example, lipopolysaccharide (LPS) preconditioning protected MSCs against apoptosis induced by hypoxia and serum deprivation *via* suppressing the extracellular signal-regulated kinase signaling pathway (Wang J. et al., 2013; Hu and Li, 2018). Preconditioning with melatonin, oxytocin, and sevoflurane also increased the resistance of MSCs to apoptosis and their paracrine activity (Mias et al., 2008; Noiseux et al., 2012; Sun et al., 2014). In addition, pretreatment with resveratrol induced autophagy in ESCs *via* activation of AMPK/ULK1 pathway (Suvorova et al., 2019). Moreover, TNF-α and other inflammatory mediators preconditioning could increase the survival, proliferation and immunomodulatory effects of MSCs and EPCs (Song et al., 2019; Beldi et al., 2020a; Ferreira et al., 2021; Nouri Barkestani et al., 2021).

### Genetic Modification

Accumulated studies have identified promising therapeutic molecular targets for genetic modification to prevent PCD of SCs. Regarding cardiovascular disease, ESCs transfected with inducible VEGF inhibited apoptosis of transplanted cell and significantly improved the cardiac function (Xie et al., 2007). In addition, overexpressing hepatocyte growth factor (HGF) modulated apoptosis of UC-MSCs and protected animals from acute liver failure (Tang et al., 2016). Also, HGF overexpression enhanced the therapeutic effect of BM-MSC for hepatocirrhosis (Zhang et al., 2018). The transduction of ERBB4 into MSCs also increased apoptotic resistance *via* activating PI3K/AKT signaling pathway (Liang et al., 2015). Moreover, HIF1α transfection improved the cardiac repair efficiency of MSCs by decreasing cardiomyocytes apoptosis (Huang et al., 2014). Importantly, the genetic upregulation of several pro-survival factors, including Bcl-2, Bcl-xl and Akt1, could increase the long-term survival of transplanted human NSCs (Korshunova et al., 2020).

### 3D Cell Culturing

Cell culture is conventionally conducted by a two-dimensional (2D) system that often does not adequately replicate the three-dimensional (3D) environment, and it is deficient in cell-to-cell interactions (Madl et al., 2018; Seo et al., 2019). The 3D culturing of bone marrow MSCs using a 3D-dynamic system exhibited decreased apoptosis and improved therapeutic effect for cardiac function (Wang Y. et al., 2018). A recently developed 3D culture clump of MSCs/extracellular matrix complexes also showed resistance against apoptosis (Komatsu et al., 2020). Furthermore, the presence of d-serine and retinoic acid in the 3D organ culture of spermatogonial SCs enhanced its therapeutic effect on spermatogenesis *via* suppressing apoptotic signaling (Modirshanechi et al., 2020). Moreover, exosomes derived from UC-MSCs under 3D culturing exerted improved osteochondral regeneration activity (Yan and Wu, 2020).

### Co-Transplantation

Co-transplantation of SC with other SCs or adult cells can also restore SCs *via* suppressing PCD. Co-transplantation of NSCs with olfactory ensheathing cells (OECs) attenuated neuronal apoptosis in traumatic brain injury (Liu et al., 2014). Also, the co-transplantation of OECs with epidermal neural crest SCs exerted a beneficial effect upon peripheral nerve injury (Zhang L. et al., 2019). Regarding repairing facial nerve axotomy, the co-transplantation of BM-MSCs and monocytes reduced apoptosis of facial nerve nucleus (Wu L. et al., 2020). Moreover, co-transplantation of ADSCs and stromal vascular fractions improved parathyroid transplantation survival *in vitro* and *in vivo* for treating hypoparathyroidism (Cui et al., 2020).

### Studies on Promoting SC Survival for Diabetes and Diabetes-Related Diseases

Regarding treating diabetes and diabetes-related diseases, multiple strategies have also been applied to improve cell survival after SC transplantation. It was reported that the hypoxic preconditioning of BM-MSCs upregulated the anti-apoptotic protein Bcl-2, thus promoting endothelial cell proliferation and decreasing the apoptosis of endothelial cells in diabetic rats (Liu et al., 2015b). Also, exposure to short-term hypoxia enhanced islet protective potential of adipose-derived MSCs (AD-MSCs) (Schive et al., 2017). In addition, hypoxia pretreatment promoted the AD-MSCs based repair of diabetic erectile dysfunction by increasing the survival of transplanted SCs in host tissues and their expression of regenerative factors (Wang et al., 2015).

Preconditioning with pharmacological or chemical agents has promoted SCs survival for treating diabetes. Preconditioning with resveratrol significantly enhanced the viability and therapeutic effect of ADSC and increased expression of the survival marker *p*-Akt for the treatment of damaged pancreas and liver dysfunction in diabetic rats (Chen et al., 2019a; Chen et al., 2019b). In addition, pretreatment with mitoTEMPO, a mitochondrial ROS scavenger, improved the survival of ADSC in diabetic mice and decreased the limb injury (Lian et al., 2019). Moreover, treatment of MSCs in combination with melatonin decreased the rate of islet cell apoptosis *via* suppressing apoptotic signaling (Kadry et al., 2018). Also, melatonin preconditioning enhanced the effect of MSCs-derived exosomes on diabetic wound healing by regulating macrophages and targeting the PTEN/AKT pathway (Liu W. et al., 2020). Notably, although metformin, the most commonly used antidiabetic drug, and BM-MSCs treatment individually improve cardiac function in diabetic cardiomyopathy, metformin can reduce the efficacy of MSCs therapy for cardiac repair during diabetic cardiomyopathy by decreasing the survival of transplanted SCs (Ammar et al., 2021).

Genetic modification improved the survival of SCs for treating diabetes and diabetes-related diseases. The overexpression of HIF1α reduced ADSC apoptosis upon high glucose conditions and enhanced the therapeutic effects on diabetic wound healing (Xu et al., 2020). The preconditioning of MSCs with deferoxamine, an iron chelator, increased the stability of HIF1α protein and homing of MSCs in streptozotocin-diabetic rats (Najafi and Sharifi, 2013). Moreover, the overexpression of AURKA promoted the effect of ADSCs on wound healing in diabetic mice *via* enhancing autophagy of ADSCs and decreasing apoptosis (Yin et al., 2020).

It was also demonstrated that co-culturing and co-transplanting of BM-MSCs and islet reduced islet destruction *in vitro* and increased anti-inflammatory effects *in vivo* (Yoshimatsu et al., 2015). Another study reported that islets co-cultured with ADSC reduced apoptosis and improved glucose-stimulated insulin secretion compared with the control group (Gamble et al., 2018). Moreover, the co-transplantation of MSCs and fetal HSCs enhanced engraftment of HSCs and promoted the therapeutic effect in T1DM (Arjmand et al., 2019).

### Implications for Future Strategies to Improve SC Therapy for Diabetes and Diabetes-Related Diseases

Although the necroptosis and ferroptosis in SCs for treating diabetic diseases are rarely reported, recent studies indicated these two types of PCD are primary mechanisms of cell death in islet transplantation, suggesting the potential value of targeting necroptosis and ferroptosis for SC therapy (Zhao et al., 2015; Yao et al., 2020). As mentioned in this review, interactions between different types of PCD also need further study and novel regulators, such as AMPK/mTOR, which coordinate multiple cell death are promising therapeutic targets to improve SC therapy for diabetes and diabetes-related diseases (Zhang et al., 2016).

Although genetic modification is an efficient method to target PCD, the use of genetic techniques raises some safety concerns (Hu C. et al., 2020). Preconditioning strategies of SCs for transplantation are an attractive alternative to overcome this potential limitation. Increased oxidative stress is considered a major factor to compromise MSCs in diabets models (Fijany et al., 2019). Evidence supporting the benefit of acute preconditioning of SCs with oxidative stress suggests that the application of preconditioning may reduce oxidative stress-induced PCD in diabetic diseases (Pendergrass et al., 2013). Moreover, preconditioning with melatonin showed a significant protective effect on SCs *via* targeting multiple types of PCD, and melatonin suppressed osteoblasts ferroptosis induced by high glucose in type 2 diabetic osteoporosis (Liu W. et al., 2020; Zhao et al., 2020b; Ma et al., 2020). Thus, melatonin is a promising agent to improve SC survival in transplantation for treating diabetes and diabetes-related diseases.

As 3D culture systems become more relevant to innate structure and physiology, the ability to adequately replicate the 3D environment experienced by transplanted SCs becomes possible. Studies of 3D cell culturing developed to reduce PCD of SCs and improve their therapeutic effects have rapidly advanced (Shimony et al., 2008; Modirshanechi et al., 2020). In addition, a 3D capacitance cell sensor has been developed to monitor cell apoptosis in real-time for 3D cell cultures (Lee et al., 2016). Although methods of 3D cell culturing of SCs that can be used for treating diabetic diseases have been established, the application of these methods and their benefit requires further exploration.

## Conclusion

Extensive and increasing evidence demonstrates that distinct types of PCD contribute to the cell death of SCs, and the inhibition of PCDs can promote the survival of SCs and their therapeutic effects in diabetes and diabetes-related diseases. These findings provide deep insights into the cell death of SCs-based therapy for diabetes and diabetes-related diseases and shed light on the future development of therapeutic strategies.
